# An Acid-Cleavable Lamellar Block Copolymer for Sub-30-nm Line Spacing Patterning via Graphoepitaxial Directed Self-Assembly and Direct Wet Etching

**DOI:** 10.3390/polym17182435

**Published:** 2025-09-09

**Authors:** Jianghao Zhan, Caiwei Shang, Muqiao Niu, Jiacheng Luo, Shengguang Gao, Zhiyong Wu, Shengru Niu, Yiming Xu, Xingmiao Zhang, Zili Li, Shisheng Xiong

**Affiliations:** 1 Center of Micro-Nano System, School of Information Science and Technology, Fudan University, Shanghai 200438, China; 21110720052@m.fudan.edu.cn (J.Z.); 24110720056@m.fudan.edu.cn (C.S.); 24110720053@m.fudan.edu.cn (M.N.); 23110720116@m.fudan.edu.cn (J.L.); 23210720229@m.fudan.edu.cn (S.N.); yimingxu23@m.fudan.edu.cn (Y.X.); lizili@fudan.edu.cn (Z.L.); 2 Zhangjiang Laboratory, 100 Haike Road, Shanghai 201204, China; gaosg@zjlab.ac.cn (S.G.); wuzy@zjlab.ac.cn (Z.W.); zhangxm@zjlab.ac.cn (X.Z.)

**Keywords:** acid-cleavable block copolymer, graphoepitaxial directed self-assembly, wet etching, line/space pattern

## Abstract

Graphoepitaxial directed self-assembly (DSA) of block copolymers (BCPs) has emerged as a promising strategy for sub-30 nm line spacing patterning in semiconductor nanofabrication. Among the available BCP systems, polystyrene-block-poly (methyl methacrylate) (PS-*b*-PMMA) has been extensively utilized due to its well-characterized phase behavior and compatibility with standard lithographic processes. However, achieving a high-fidelity pattern with PS-*b*-PMMA remains challenging, owing to its limited etch contrast and reliance on UV-assisted degradation for PMMA removal. In this study, we report the synthesis of an acid-cleavable lamellar BCP, PS-N=CH-PMMA, incorporating a dynamic Schiff base (-N=CH-) linkage at the junction. This functional design enables UV-free wet etching, allowing selective removal of PMMA domains using glacial acetic acid. The synthesized copolymers retain the self-assembly characteristics of PS-*b*-PMMA and form vertically aligned lamellar nanostructures, with domain spacings tunable from 36.1 to 40.2 nm by varying the PMMA block length. When confined within 193i-defined trench templates with a critical dimension (CD) of 55 nm (trench width), these materials produced well-ordered one-space-per-trench patterns with interline spacings tunable from 15 to 25 nm, demonstrating significant line spacing shrinkage relative to the original template CD. SEM and FIB-TEM analyses confirmed that PS-N=CH-PMMA exhibits markedly improved vertical etch profiles and reduced PMMA residue compared to PS-*b*-PMMA, even without UV exposure. Furthermore, Ohta–Kawasaki simulations revealed that trench sidewall angle critically influences PS distribution and residual morphology. Collectively, this work demonstrates the potential of dynamic covalent chemistry to enhance the wet development fidelity of BCP lithography and offers a thermally compatible, UV-free strategy for sub-30 nm nanopatterning.

## 1. Introduction

As integrated circuit (IC) manufacturing advances toward the 2 nm technology node, the continued miniaturization of device architectures and increasing interconnect complexity impose stringent demands on the fabrication of high-resolution sub-30 nm line/space patterns [1,2,3]. Deep ultraviolet (DUV) lithography based on 193 nm exposure has been extensively adopted in advanced logic fabrication due to its mature process integration and compatibility with existing production lines [4,5]. However, overcoming the optical diffraction limit in DUV lithography typically requires double or multiple patterning techniques, which significantly increase process complexity, cost, and defect density [6,7]. Extreme ultraviolet (EUV) lithography, utilizing a 13.5 nm light source, offers superior resolution and has enabled single-exposure fabrication of line/space patterns with widths ranging from 13 nm to 16 nm in several critical layers [5,8]. Nevertheless, the high capital cost, sensitivity to particle contamination, and challenges in fab-level implementation continue to hinder its widespread adoption in middle-of-line (MOL) layers such as interconnects [9,10]. Accordingly, the development of scalable patterning strategies compatible with existing DUV platforms remains of great engineering significance. Among them, the combination of DUV lithography with directed self-assembly (DSA) has attracted increasing attention for its ability to synergistically integrate top-down pattern control with bottom-up resolution enhancement [11].

DSA of BCPs, a bottom-up patterning technique, has been widely recognized as a promising complementary lithography method due to its low cost, scalability, and high resolution [12,13]. DSA leverages the microphase separation of BCPs within pre-patterned templates to generate periodic nanostructures with precisely controllable dimensions [14,15]. In particular, graphoepitaxial DSA of lamella-forming BCPs in trench array templates enables aligned line/space arrays with tunable pitch and critical dimensions [16,17]. These nanoscale features are suitable for fabricating densely packed FinFET fins [18], advanced gate structures [19], and subwavelength photonic components [20]. Among various BCPs, polystyrene-block-poly(methyl methacrylate) (PS-*b*-PMMA) has been widely employed in DSA applications due to its well-established phase behavior [21], facile synthesis [22], and excellent process compatibility with standard lithographic processes [23]. However, this system suffers from low etch contrast between PS and PMMA during pattern formation, particularly during the PMMA removal step for PS mask fabrication, which limits pattern fidelity and narrows the process window [24,25]. Two main strategies are currently employed to remove the PMMA block in PS-*b*-PMMA systems: dry etching and wet etching [26,27]. Dry etching typically involves oxygen plasma in reactive ion etching (RIE) or inductively coupled plasma (ICP) systems to achieve anisotropic removal of polymer materials [28,29]. The etch selectivity between PMMA and PS is generally low (typically 2:1 to 4:1), leading to PS mask erosion, line edge blurring, and pattern distortion, which hinder the retention of high-fidelity structures [30,31]. In contrast, wet etching offers milder processing conditions and superior material selectivity, resulting in improved interface cleanliness and pattern control [32]. Conventional wet etching strategies usually require ultraviolet (UV) pre-exposure to induce chain scission in the PMMA domains, followed by selective dissolution in weak acidic solvents such as glacial acetic acid to construct PS masks [33]. UV-assisted processes often suffer from issues such as nonuniform photochemical reactions, residual PMMA at the PS/PMMA interface, and undesired crosslinking of the PS phase, thereby compromising pattern integrity and narrowing the process window [34]. These challenges highlight the urgent need for structurally modified PS-*b*-PMMA analogues that incorporate cleavable junctions to enable UV-free PMMA removal and high-fidelity pattern transfer during wet development. Recent developments in dynamic covalent chemistry have introduced versatile tools for designing stimuli-responsive polymers [35]. Among them, Schiff base linkages (-N=CH-), formed via condensation between aromatic aldehydes and primary amines [36], exhibit rapid acid-induced cleavage under mild conditions [37] and have been extensively studied in degradable polymers [38], drug delivery systems [39], and responsive materials [40]. These bonds offer controllable reaction conditions and yield structurally stable intermediates [41]. Recent advances have highlighted the utility of dynamic covalent chemistry in nanofabrication [42]. In BCP design, preliminary studies have explored the use of Schiff base-cleavable junctions to impart acid sensitivity [43,44,45]. To the best of our knowledge, such linkages have not yet been applied to PS-*b*-PMMA systems, nor have they been evaluated in the context of DSA and wet etching processes. Incorporating Schiff base-cleavable linkages into the BCP backbones may enable UV-free selective removal of the PMMA domain, while minimizing interfacial PMMA residue at the interface and enhancing pattern fidelity during etching.

**Scheme 1:**
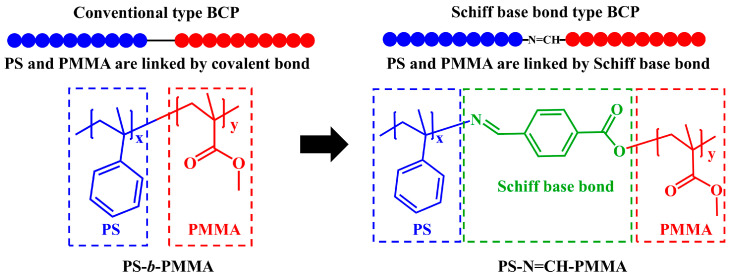
Concept and chemical structure of conventional type PS-*b*-PMMA and Schiff base bond type PS-N=CH-PMMA.

Here, for the first time, we designed and synthesized a novel acid-cleavable lamella-forming block copolymer, PS-N=CH-PMMA, by introducing a Schiff base (benzylideneimine) linkage between the PS and PMMA segments (as illustrated in Scheme 1). This structural design imparts acid sensitivity to the copolymer, enabling UV-free selective wet etching under mildly acidic conditions. Meanwhile, the material retains the desirable thin film self-assembly characteristics of conventional PS-*b*-PMMA, including the formation of well-ordered nanostructures upon thermal annealing at 230 °C and strong compatibility with graphoepitaxial directed self-assembly (DSA) processes. PS-N=CH-PMMA forms vertically oriented lamellar structures in thin films, exhibiting fingerprint-like morphologies. By tuning the molecular weight of the aldehyde-functionalized PMMA precursor (PMMA-CHO), both the domain spacing (*L*_0_) of the self-assembled films and the critical dimensions of the DSA-generated patterns can be effectively modulated. When assembled within trench templates fabricated via 193 nm DUV lithography, the resulting line/space arrays exhibit interline space widths ranging from 15 to 25 nm. Upon treatment with glacial acetic acid, the PMMA domains are selectively removed, yielding well-defined PS masks with significantly enhanced pattern fidelity. Following wet etching, the cross-sectional profiles of the DSA patterns were characterized by focused ion beam transmission electron microscopy (Fib-TEM), confirming etch depths of up to 93.3 nm, thereby demonstrating excellent pattern retention and etch compatibility. Furthermore, Ohta–Kawasaki (OK) simulations reveal that steep sidewalls in the guiding templates can promote PS retention after etching, highlighting the critical role of template geometry in determining the residual morphology. Compared to conventional PS-*b*-PMMA, PS-N=CH-PMMA maintains robust self-assembly performance while exhibiting markedly improved responsiveness to wet etching, thereby validating the effectiveness of the functional design. This study presents a viable molecular design strategy for next-generation UV-free lithography, offering a simplified, high-fidelity approach to nanoscale pattern fabrication.

## 2. Materials and Methods

### 2.1. Materials

Ethyl α-bromophenylacetate (EBPA), 2-hydroxyethyl 2-bromo-2-methylpropanoate (HEBIB), 4-dimethylaminopyridine (DMAP), 1-(3-dimethylaminopropyl)-3-ethylcarbodiimide hydrochloride (EDC·HCl), DCM (dichloromethane), anhydrous ethanol, neutral alumina (200–300 mesh), and 4-formylbenzoic acid were obtained from Aladdin. Cuprous bromide (CuBr), anhydrous diethyl ether, propylene glycol methyl ether acetate (PGMEA), anhydrous methanol, tetrahydrofuran (THF), N, N, N′, N″, N″-pentamethyldiethylenetriamine (PMDETA), styrene (St), methyl methacrylate (MMA), glycidyl methacrylate (GMA), anisole, and glacial acetic acid (electronic grade, ≥99.7%) were sourced from Sinopharm Chemical Reagent Co., Ltd., Shanghai, China. St and MMA were passed through neutral alumina columns to remove inhibitors prior to use. CuBr was purified by washing with dilute acetic acid, followed by rinsing with acetone and drying under vacuum, and then stored under a nitrogen atmosphere. All other solvents and reagents were used without further purification. Silicon wafers were provided by Suzhou Research Material Micro Nano Technology Co., Ltd, Suzhou, China. Amino-terminated polystyrene (PS-NH_2_, M_n_ = 32 kg/mol, PDI = 1.07) and hydroxyl-terminated polystyrene (PS-OH; M_n_ = 8 kg/mol; PDI = 1.09) were purchased from Polymer Source Inc., Dorval, QC, Canada. The guiding templates with periodic trench arrays for DSA were fabricated via 193i lithography and kindly supplied by the Shanghai IC R&D Center (ICRD), Shanghai, China.

### 2.2. Syntheses of Polymers

#### 2.2.1. Synthesis of Bromide-Terminated Poly (Methyl Methacrylate) (PMMA_28k_-Br)

PMMA_28k_-Br was synthesized via atom transfer radical polymerization (ATRP) using EBPA as the initiator, following a modified literature procedure [46]. MMA (200 mL, 1.88 mol), anisole (200 mL), EBPA (0.972 g, 4.0 mmol), and PMDETA (0.720 g, 4.16 mmol) were sequentially introduced into a thoroughly dried 500 mL Schlenk flask under nitrogen atmosphere. The reaction mixture was degassed by purging with nitrogen gas for 1 h to remove dissolved oxygen. Subsequently, CuBr (0.574 g, 4.0 mmol) was quickly added under a nitrogen atmosphere, and the reaction solution was stirred at ambient temperature for 30 min to ensure homogeneous formation of the catalyst complex. The polymerization was initiated by placing the flask in an oil bath at 60 °C and was allowed to proceed for 2.5 h. The reaction was quenched by rapid cooling in liquid nitrogen. The resultant polymer solution was diluted with THF and passed through a neutral alumina column to remove residual copper catalyst. The purified polymer was precipitated into excess anhydrous methanol. The white solid product was collected by filtration, thoroughly washed with methanol, and dried under vacuum at 40 °C for 48 h. ^1^H NMR (400 MHz, CDCl_3_) δ: 0.80–1.10 ppm (m, -CH_3_), 1.80–2.00 ppm (m, -CH_2_- backbone), 3.60 ppm (s, -OCH_3_).

#### 2.2.2. Synthesis of Polystyrene-Block-Poly (Methyl Methacrylate) (PS_28k_-*b*-PMMA_28k_)

The synthesis of PS_28k_-*b*-PMMA_28k_ via ATRP was conducted following previously reported protocols with minor modifications [47]. PMMA_28k_-Br (28 g) was dissolved in anisole (250 mL) and styrene (125 mL) at 40 °C under stirring to ensure complete dissolution. The solution was transferred into a 500 mL Schlenk flask and deoxygenated by nitrogen bubbling for 1 h. PMDETA (0.174 g) and CuBr (0.143 g) were then added under a nitrogen atmosphere, followed by an additional 30 min of purging. Polymerization was initiated by heating the mixture to 100 °C in an oil bath. Aliquots were periodically withdrawn for GPC analysis to monitor molecular weight evolution. Upon reaching the target degree of polymerization, the reaction was quenched by immersion in liquid nitrogen. The mixture was diluted with THF and passed through a neutral alumina column to remove residual copper salts. The product was precipitated into anhydrous methanol, and fractional precipitation using a THF–methanol solvent pair was employed to remove unreacted PMMA. The resulting white solid was washed with cyclohexane to eliminate residual PS and dried under vacuum at 60 °C for 48 h. ^1^H NMR (400 MHz, CDCl_3_) δ: 0.80–1.10 ppm (m, -CH_3_), 1.80–2.00 ppm (m, -CH_2_- backbone), 3.60 ppm (s, -OCH_3_ of PMMA), and 6.30–7.20 ppm (m, Ar-H, aromatic protons of PS).

#### 2.2.3. Synthesis of Hydroxy-Terminated Poly (Methyl Methacrylate) (PMMA-OH)

Hydroxy-terminated PMMA samples with target M_n_ values of 23k, 26k, 28k, and 30k were prepared via a modified procedure based on previously reported protocols [48]. Briefly, HEBIB (0.21 g), PMDETA (0.17 g), MMA (100 mL), and anisole (200 mL) were added to a 500 mL Schlenk flask. The solution was degassed by nitrogen bubbling for 1 h, after which CuBr (0.14 g) was added under a nitrogen atmosphere. Following an additional 30 min of purging, aliquots were collected at predetermined time points during polymerization at 60 °C polymerization to monitor molecular weight development via GPC. The reaction was terminated by quenching with liquid nitrogen. The mixture was diluted with THF and passed through a neutral alumina column to remove copper salts. The resulting solution was precipitated in anhydrous methanol, and the crude product was collected by filtration. Further purification was achieved by redissolving the polymer in THF and reprecipitating in methanol twice. The final product was dried under vacuum at 40 °C for 48 h. ^1^H NMR (400 MHz, CDCl_3_) δ: 0.80–1.10 (m, -CH_3_), 1.80–2.00 (m, -CH_2_- backbone), 3.60 (s, -OCH_3_ of PMMA), 4.10 (t, -CH_2_OH). The number-average degree of polymerization (DP_n_) of the PMMA backbone was estimated based on GPC results using PMMA standards and calculated according to Equation (1):

(1) $${DP}_{n}=\frac{M_{n}}{100.12}\times 100\;\%$$

#### 2.2.4. Synthesis of Aromatic Aldehyde-Terminated Poly (Methyl Methacrylate) (PMMA-CHO)

A series of aldehyde-functionalized PMMA (PMMA-CHO) samples were synthesized via Steglich esterification, following established procedures for ester coupling reactions [49], using PMMA-OH precursors with M_n_ values of 23k, 26k, 28k, and 30k. The following procedure describes the synthesis route using PMMA_28k_-OH as a representative example. PMMA28k-OH (0.28 g), DMAP (0.32 g), EDC·HCl (1.92 g), and 4-formylbenzoic acid (12.23 g) were placed in a 250 mL round-bottom flask. Dichloromethane (100 mL) was then added to the mixture. The reaction was carried out at 35 °C with magnetic stirring for 72 h. Upon completion, the mixture was precipitated in anhydrous ether, yielding a white solid. This solid was subsequently washed several times with a mixture of tetrahydrofuran and anhydrous methanol to remove any unreacted materials and impurities. The final purified product was dried in a vacuum oven at 40 °C for 24 h to eliminate any residual solvents. ^1^H NMR (400 MHz, CDCl_3_) δ: 0.80–1.10 (m, -CH_3_), 1.80–2.00 (m, -CH_2_- backbone), 3.60 (s, -OCH_3_ of PMMA), 7.90–8.10 (d, Ar-H), and 10.00 (s, -CHO). The end-group functionalization efficiency of PMMA–CHO was quantitatively determined by ^1^H NMR spectroscopy. Specifically, the characteristic signal of the aldehyde proton (-CHO) appeared as a singlet at δ ≈ 10.0 ppm and was integrated relative to the methoxy protons (-OCH_3_) of the PMMA repeating units, which typically appear at δ ≈ 3.6 ppm (3H per repeat unit). The grafting efficiency (GE) of the aldehyde groups was calculated using the following Equation (2):

(2) $$GE\left(\%\right)=\left(\frac{{3A}_{CHO}}{A_{OCH_{3}}}\right)\times DP_{n}\times 100\;\%$$

where $A_{CHO}\;$is the integral of the aldehyde proton peak, and $A_{OCH_{3}}\;$is the total integral of the methoxy proton signal. The factor of 3 accounts for the three protons per methoxy group. This calculation assumes that each PMMA chGEain contains a single -CHO group at the α-chain terminus.

#### 2.2.5. Synthesis of Acid-Cleavable Polystyrene-Block-Poly (Methyl Methacrylate) (PS-N=CH-PMMA)

Acid-cleavable lamella-forming BCPs, PS-N=CH-PMMA, were synthesized via Schiff base condensation between amino-terminated polystyrene (PS_32k_-NH_2_) and aldehyde-functionalized PMMA (PMMA-CHO), following previously reported procedures with minor modifications. As a representative example, PS_32k_-N=CH-PMMA_28k_ was synthesized by dissolving PMMA_28k_-CHO (0.28 g) and PS_32k_-NH_2_ (0.32 g) in 20 mL of tetrahydrofuran (THF) in a 50 mL round-bottom flask. The reaction mixture was stirred at 35 °C for 24 h to allow complete Schiff base formation. The resulting product was precipitated into anhydrous ether and further purified by fractional precipitation using a THF–methanol solvent pair to remove unreacted PMMA-CHO. The precipitate was then washed with cyclohexane to eliminate any residual PS-NH_2_ and dried under vacuum at 40 °C for 48 h to afford the final PS_32k_-N=CH-PMMA_28k_ product. The volume fraction of PS (ƒ_St_) was calculated according to literature-reported methods based on block molecular weights and the bulk densities of PS (1.05 g/cm^3^) and PMMA (1.18 g/cm^3^) [50]. ^1^H NMR (400 MHz, CDCl_3_) δ: 0.80–1.10 (m, -CH_3_), 1.80–2.00 (m, -CH_2_- backbone), 3.60 (s, -OCH_3_ of PMMA), 6.30–7.20 (m, Ar-H of PS), and 7.54 (s, -N=CH-).

#### 2.2.6. Synthesis of the Random Copolymer PS-r-PMMA-r-PGMA

Poly (St-co-MMA-co-GMA) (Mat) random copolymers were synthesized via free-radical solution polymerization, with varying styrene molar fractions (F_St_). The synthesis of Mat63 (F_St_ = 63%) is described here as a representative example. St (63 mmol, 6.56 g), MMA (35 mmol, 3.51 g), and GMA (2 mmol, 0.28 g) were dissolved in 20 mL of anisole in a 50 mL round-bottom flask. AIBN (0.5 mmol, 0.082 g) was added as the thermal initiator. The solution was degassed by nitrogen bubbling at room temperature for 1 h to remove dissolved oxygen, after which polymerization was carried out at 70 °C for 8 h under a nitrogen atmosphere. After completion, the reaction mixture was diluted with THF and precipitated dropwise into excess anhydrous methanol. The resulting polymer was collected by filtration, redissolved in THF, and reprecipitated in methanol three times to remove unreacted monomers and impurities. The final white powder was obtained by drying the purified product under vacuum at 40 °C until the weight was constant. The styrene molar fraction (F_st_) in the random copolymers was calculated following the method described in reference [51]. ^1^H NMR (400 MHz, CDCl_3_) δ: 0.80–1.10 (m, -CH_3_), 1.80–2.00 (m, -CH_2_- backbone), 2.50–3.60 (m, -OCH_3_ of MMA and epoxide protons of GMA), 6.30–7.20 (m, Ar-H).

### 2.3. Thermal Annealing and Wet Etching Processes

#### 2.3.1. Thin Film Self-Assembly of BCPs

Silicon wafers (1 cm × 1 cm) were sequentially cleaned by ultrasonication in ethanol, acetone, and isopropyl alcohol for 5 min each. Afterwards, the substrates were treated with oxygen plasma for 5 min using a CPC-B plasma cleaner to remove residual organic contaminants and convert surface -Si-CH_3_ groups into hydrophilic -Si-OH functionalities, thereby improving the adhesion of the subsequent polymer layers. Random copolymers (Mat) with styrene molar fractions of 63%, 65%, 67%, and 72% (denoted as M63, M65, M67, and M72) were dissolved in toluene with a mass fraction of 0.5 wt%, and the resulting solutions were spin-coated onto the plasma-treated silicon substrates at 2000 rpm. The films were then annealed at 250 °C for 10 min under vacuum to promote thermal crosslinking of the glycidyl groups and enable covalent grafting of the Mat layer to the substrate. Non-grafted Mat polymers were removed by ultrasonication in toluene for 5 min, resulting in Mat-grafted substrates with varying PS volume fractions. Subsequently, PS-N=CH-PMMA and PS-*b*-PMMA BCPs were separately dissolved in PGMEA with a mass fraction of 2.0 wt%, and the solutions were spin-coated onto the Mat-modified substrates at 2000 rpm. The films were subsequently annealed at 230 °C for 30 min under vacuum to promote thermally driven microphase separation, leading to the formation of fingerprint-like lamellar nanostructures characteristic of lamellar-phase BCP self-assembly. All experiments were repeated three times.

#### 2.3.2. Graphoepitaxial Directed Self-Assembly of BCPs

Graphoepitaxial trench templates (1 cm × 1 cm), fabricated via 193i lithography, were provided by the Shanghai IC R&D Center. The template wafers were first cleaned by ultrasonication in isopropyl alcohol for 2 min to remove surface contaminants. A polymer brush layer was then applied by spin coating a 0.5 wt% solution of PS-OH in PGMEA at 2000 rpm onto the trench-patterned substrates. The samples were subsequently annealed in a vacuum annealing furnace at 220 °C for 10 min to enable the formation of a PS-affinitive guiding layer via the grafting of PS-OH within the trenches. Residual ungrafted PS-OH was removed by rinsing with PGMEA. Following surface functionalization, BCP thin films were prepared by spin coating 1.5 wt% solutions of either PS-*b*-PMMA or PS-N=CH-PMMA in PGMEA onto the brush-modified guiding substrates at 2000 rpm. A final annealing step was performed at 230 °C for 30 min under vacuum to induce microphase separation and achieve confined self-assembly of the BCPs within the trench structures, resulting in the formation of well-aligned line/space patterns. All experiments were repeated three times.

#### 2.3.3. Wet Etching Process

The wet etching process was performed by directly immersing the self-assembled and directed self-assembled samples (1 cm × 1 cm) into electronic-grade glacial acetic acid for 5 min. After etching, the samples were promptly blown dry with a stream of nitrogen gas.

### 2.4. Ohta–Kawasaki (OK) Model Simulation

To investigate the microphase separation behavior of lamellar PS_32k_-N=CH-PMMA_28k_ (*L*_0_ = 38.3nm) under graphoepitaxial confinement with varying trench sidewall angles during the pattern-shrinkage process, three-dimensional simulations were performed using the OK model, following the method reported by Yoshimoto et al. [52]. The simulation domain was discretized into a cubic grid with a spatial resolution of Δ*l* = 0.2 *R_g_* and periodic boundary conditions applied in all directions. The trench template was defined with a normalized depth of 5.26 *L*_0_, width of 1.45 *L*_0_, and length of 3 *L*_0_. The sidewalls and bottom surface of the trench were set to be preferential to PS by applying a positive surface interaction parameter *Λ*(*r*), while the top surface remained neutral.

### 2.5. Characterization

The proton nuclear magnetic resonance (^1^H NMR) spectra were obtained using a Bruker Avance NEO 400 MHz NMR spectrometer, with chloroform-d as the solvent. Fourier transform infrared (FTIR) spectra for these polymers were acquired using a Thermo Nicolet iS50 FTIR spectrometer ( Thermo Fisher Scientific, Waltham, MA, USA), recorded over the range of 400–4000 cm^−1^, with a resolution of 4 cm^−1^ and 32 scans per measurement. The molecular weights and polydispersity indices (PDI) of all polymer samples were measured by gel permeation chromatography (GPC) on an Agilent 1260 system (Agilent Technologies, Santa Clara, CA, USA), using tetrahydrofuran (THF) as the eluent at a flow rate of 1 mL/min and calibrated with polystyrene standards. Differential scanning calorimetry (DSC) analyses were carried out using a Netzsch DSC 200 F3 instrument (NETZSCH-Gerätebau GmbH, Selb, Germany), within a temperature range of 30 to 200 °C at a heating rate of 20 °C/min during the second heating cycle. To mitigate the influence of thermal history, DSC heating and cooling tests were repeated three times. Thermogravimetric analysis (TGA) was performed with a Mettler SDTA 851e instrument (Mettler-Toledo GmbH, Greifensee, Switzerland), heating samples from 30 °C to 800 °C under a nitrogen atmosphere at a rate of 20 °C/min. The morphology of the samples was examined using a field-emission scanning electron microscope (FE-SEM, Gemini 300, Carl Zeiss Microscopy GmbH, Oberkochen, Germany) in their as-received state without gold coating. The film thicknesses were determined using a Filmetrics F20-UV reflectometer (KLA Corporation, Milpitas, CA, USA), which utilizes broadband spectroscopic reflection over a 190–1100 nm wavelength range. Assuming a refractive index of 1.46, the system can measure thicknesses between 5 nm and 40 μm with a resolution of 0.02 nm. Focused ion beam transmission electron microscopy (FIB-TEM) analyses were performed at Nanjing Pan Quan Electronic Technology Co., Ltd. (Nanjing, China), with a protective hafnium oxide layer deposited on the sample surface prior to FIB preparation. TEM samples were prepared using a commercial Helios DualBeam system (Thermo Fisher Scientific, Waltham, MA, USA). TEM imaging was performed with a Talos transmission electron microscope (Thermo Fisher Scientific, Waltham, MA, USA), operated at an accelerating voltage of 200 kV, and images were acquired in bright-field mode.

## 3. Results and Discussion

### 3.1. Synthesis and Characterization of Precursor Polymers and BCPs

**Figure 1:**
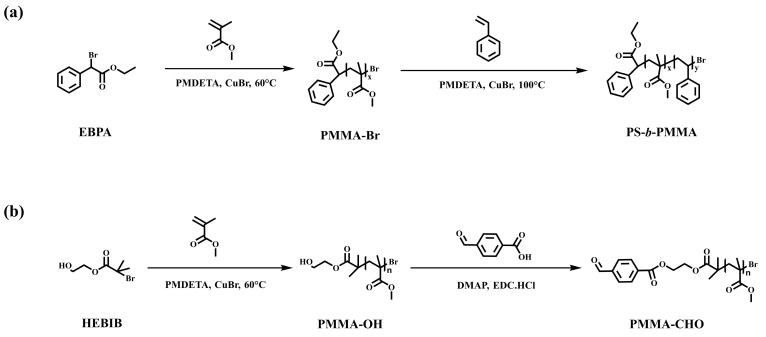

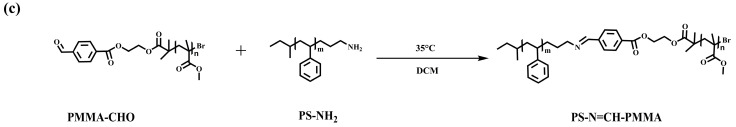
Synthetic routes for (**a**) conventional BCP PS-*b*-PMMA, (**b**) aldehyde-terminated PMMA (PMMA-CHO), and (**c**) acid-cleavable BCP PS-N=CH-PMMA.

The synthetic routes for the conventional BCP PS-*b*-PMMA and the acid-cleavable analogue PS-N=CH-PMMA are illustrated in Figure 1. As shown in Figure 1a, PS-*b*-PMMA was synthesized via a sequential atom transfer radical polymerization (ATRP) strategy. Initially, PMMA-Br was prepared by polymerizing methyl methacrylate (MMA) using ethyl 2-bromophenylacetate (EBPA) as the initiator in the presence of CuBr and PMDETA at 60 °C. The resultant PMMA macroinitiator was then chain-extended with styrene at 100 °C to afford the desired PS-*b*-PMMA BCP. The aldehyde-terminated PMMA (PMMA-CHO) precursor was synthesized, as outlined in Figure 1b. 2-Hydroxyethyl 2-bromoisobutyrate (HEBIB) was employed as the ATRP initiator to yield hydroxyl-terminated PMMA (PMMA-OH). The terminal hydroxyl group was subsequently esterified with 4-formylbenzoic acid using a carbodiimide-mediated coupling strategy to yield the desired PMMA-CHO precursor. In this reaction, EDC·HCl served as the coupling agent to activate the carboxylic acid into an O-acylisourea intermediate, while DMAP acted as a nucleophilic catalyst to facilitate acyl transfer [53]. The final BCP PS-N=CH-PMMA was obtained by condensation of PMMA-CHO with amino-terminated polystyrene (PS-NH_2_) in dichloromethane at 35 °C, forming a dynamic Schiff base linkage at the block junction (Figure 1c).

**Figure 2:**
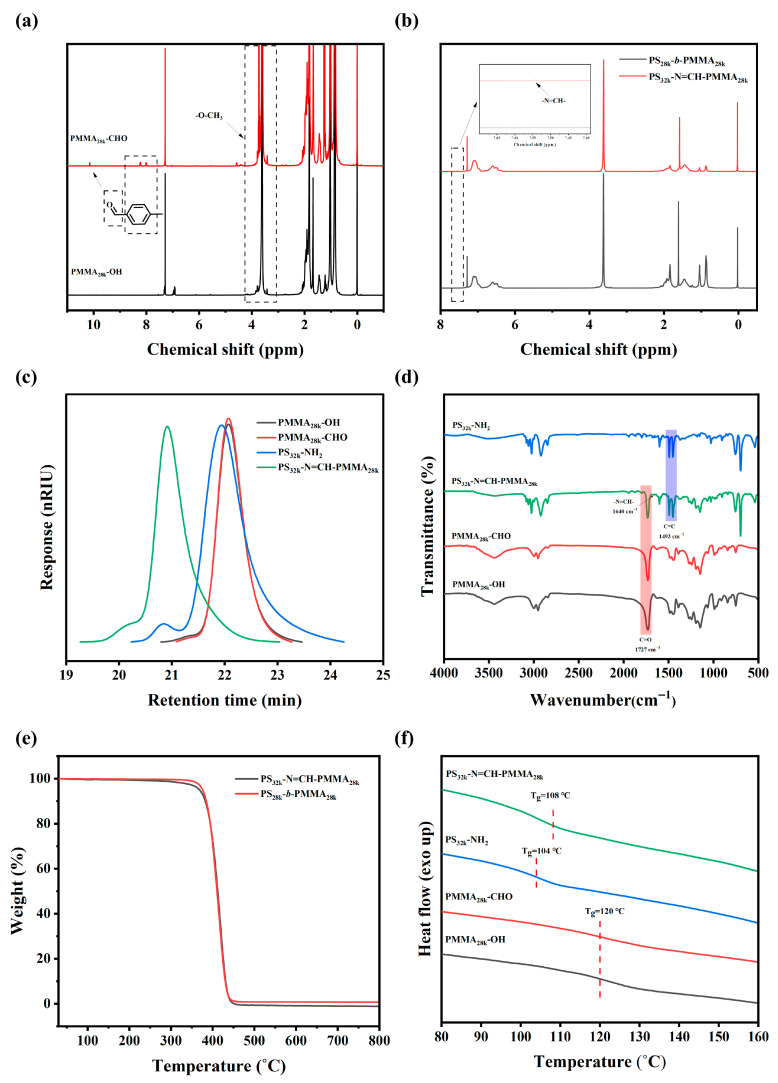
Characterization of precursor polymers and BCPs. (**a**) ^1^H NMR spectra of PMMA_28k_-OH and PMMA_28k_-CHO. (**b**) ^1^H NMR spectra of PS_28k_-*b*-PMMA_28k_ and PS_32k_-N=CH-PMMA_28k_. (**c**) GPC traces of PMMA_28k_-OH, PMMA_28k_-CHO, PS_32k_-NH_2_, and PS_32k_-N=CH-PMMA_28k_. (**d**) FTIR of PMMA_28k_-OH, PMMA_28k_-CHO, PS_32k_-NH_2_, and PS_32k_-N=CH-PMMA_28k_. (**e**) TGA curves of PS_28k_-*b*-PMMA_28k_ and PS_32k_-N=CH-PMMA_28k_. (**f**) DSC thermograms of PMMA_28k_-OH, PMMA_28k_-CHO, PS_32k_-NH_2_, and PS_32k_-N=CH-PMMA_28k_.

Figure 2 summarizes the comprehensive characterization of the precursor polymers and the resulting BCPs. As shown in Figure 2a, the ^1^H NMR spectrum of PMMA_28k_-OH exhibits a distinct methoxy proton (-OCH_3_) signal at δ = 3.6 ppm, along with broad backbone peaks between δ = 0.8 and 2.0 ppm. Upon end-functionalization with 4-formylbenzoic acid, the aldehyde-terminated PMMA_28k_-CHO displays a characteristic aromatic proton signal at δ = 7.9–8.0 ppm and the aldehydic proton resonance at δ = 10.0 ppm, confirming successful introduction of the aldehyde group [54]. Figure 2b presents the ^1^H NMR spectra of the conventional BCP PS_28k_-*b*-PMMA_28k_ and the acid-cleavable analogue PS_32k_-N=CH-PMMA_28k_. A characteristic imine proton signal at δ = 7.54 ppm, which is absent in the spectrum of PS_28k_-*b*-PMMA_28k_, confirms the successful formation of the Schiff base linkage at the junction between the two blocks [55]. ^1^H NMR spectra of PS_32k_-N=CH-PMMA BCPs with different PMMA block lengths are provided in Figure S1. Figure 2c shows the GPC traces of PMMA_28k_-OH, PMMA_28k_-CHO, PS_32k_-NH_2_, and the resulting BCP PS_32k_-N=CH-PMMA_28k_. GPC traces of hydroxyl- and aldehyde-terminated PMMA precursors with targeted molecular weights of 23k, 26k, 28k, and 30k are provided in Figures S2 and S3. Compared to the individual precursors, PS_32k_-N=CH-PMMA_28k_ exhibits a clear shift toward shorter retention time, consistent with an increase in molecular weight following BCP formation via imine linkage. Additional GPC traces of PS_32k_-N=CH-PMMA BCPs are provided in Figure S4. Similarly, the GPC profiles of PMMA_28k_-Br and its chain-extended product PS_28k_-*b*-PMMA_28k_ are shown in Figure S5. The GPC data of the precursor polymers are summarized in Table 1 and Table 2,while the GPC and ^1^H NMR characterization results of the resulting BCPs are provided in Table 3. The chemical bonding features of the copolymers were further investigated via FTIR spectroscopy. Figure 2d shows the FTIR spectra of PMMA_28k_-OH, PMMA_28k_-CHO, PS_32k_-NH_2_, and PS_32k_-N=CH-PMMA_28k_. The spectrum of PS_32k_-N=CH-PMMA_28k_ exhibits a combination of characteristic bands from both precursor segments, including a strong absorption at 1727 cm^−1^ attributed to the C=O stretching of the ester groups in the PMMA block, and a characteristic band at 1493 cm^−1^ corresponding to the aromatic C=C stretching of the benzene ring in the PS block. Notably, a new peak appears at 1640 cm^−1^, which is absent in the spectra of the precursors and can be assigned to the imine stretching vibration, confirming the successful synthesis of the acid-cleavable BCP. The thermal stability and glass transition behavior of the synthesized BCPs were evaluated by TGA and DSC, as shown in Figure 2e,f. In the TGA profiles (Figure 2e), the acid-cleavable PS_32k_-N=CH-PMMA_28k_ and the conventional PS_28k_-*b*-PMMA_28k_ exhibit nearly identical thermal stability, with no significant weight loss observed below 250 °C. This thermal robustness confirms their compatibility with typical thermal annealing protocols used in DSA processes [56]. The DSC thermogram presented in Figure 2f displays a single glass transition temperature (T_g_) at 108 °C for PS_32k_-N=CH-PMMA_28k_, which lies between the T_g_ values of the corresponding PS_32k_-NH_2_ (104 °C) and PMMA_28k_-CHO (120 °C) precursor polymers. Unlike conventional PS-*b*-PMMA systems, which typically exhibit two distinct T_g_s, the single T_g_ observed here may result from the similar segment lengths and closely spaced intrinsic T_g_ values of the PS and PMMA blocks, leading to overlapping thermal transitions [57].

### 3.2. Morphological Analysis of BCP Self-Assembled Thin Films After Wet Etching

**Figure 3:**
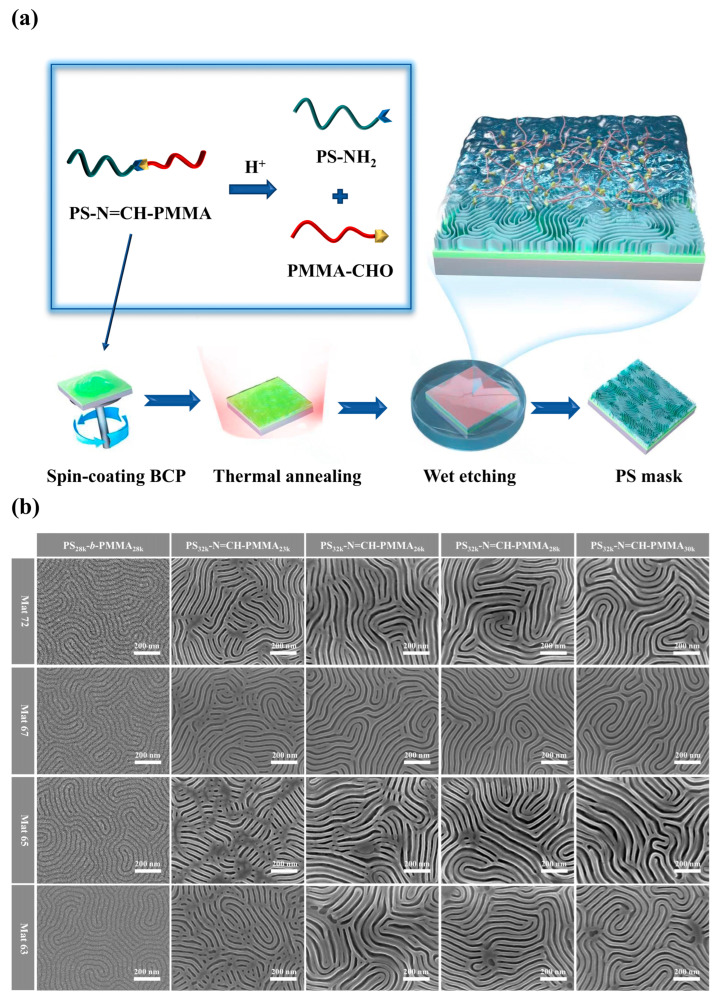
Lamella-forming PS-N=CH-PMMA BCP thin film self-assembly and wet etching for PS mask fabrication. (**a**) Schematic illustration of the process for fabricating PS masks from acid-cleavable PS-N=CH-PMMA BCP. (**b**) Top-view SEM images of PS_28k_-*b*-PMMA_28k_ and a series of PS_32k_-N=CH-PMMA_x_ (x = 23k, 26k, 28k, and 30k) thin films thermally annealed on different mats (Mat 63, Mat 65, Mat 67, and Mat 72), followed by wet etching.

To evaluate the thin film self-assembly behavior and etching responsiveness of the PS-N=CH-PMMA BCP, we prepared a series of lamella-forming samples. The PMMA block length was systematically varied, and the samples were formed on substrates coated with different neutral mats. As confirmed by the thermal analysis in Figure 2e, this acid-labile material remains thermally stable below 250 °C, which enables conventional thermal annealing without cleavage of the imine linkage. Accordingly, the BCP films were annealed at 230 °C, a representative processing temperature for lamella-forming PS-*b*-PMMA systems [56]. The BCP film thickness was controlled at approximately 1.25 *L*_0_ to promote perpendicular lamellar orientation under favorable confinement conditions [58,59]. As schematically illustrated in Figure 3a, the mask fabrication process consists of three key steps. A thin film was prepared by spin coating the PS-N=CH-PMMA BCP solution onto the neutral mat layer, followed by thermal annealing to induce microphase separation and generate fingerprint-like lamellar morphologies. The resulting film is then immersed in dilute acetic acid, during which the imine linkage is hydrolyzed and the PMMA domains are selectively removed via a single-step wet etching process. This UV-free approach leverages the acid-cleavable nature of the -N=CH- linkage to trigger domain separation under mild chemical conditions, eliminating the need for high-energy exposure steps.

In contrast to conventional PS-b-PMMA systems that require UV exposure to render PMMA domains soluble, the acid-cleavable PS-N=CH-PMMA obviates this step. It enables direct wet etching without any UV pretreatment and yields well-defined PS mask patterns with sharp contours. Figure 3b compares the top-view SEM morphologies of PS_28k_-*b*-PMMA_28k_ and a series of PS_32k_-N=CH-PMMA_x_ (x = 23k, 26k, 28k, 30k) thin films after thermal annealing and subsequent UV-free wet etching on four different neutral mats (Mats 63–72). The four random copolymers were synthesized via free-radical copolymerization, and the detailed synthetic procedures are provided in Figures S6–S8. The molecular weights, styrene molar fractions (F_St_), and film thicknesses of the random copolymers are summarized in Table 4. The PS_28k_-*b*-PMMA_28k_ exhibits well-defined lamellae after annealing but shows very limited contrast after etching, indicating ineffective domain-selective removal in the absence of UV exposure. This suggests that the covalent linkage between PS and PMMA blocks hinders selective etching when no photolysis is employed. In contrast, all PS-N=CH-PMMA samples exhibit clear domain contrast and well-preserved lamellar structures, confirming efficient and UV-free PMMA removal through acid-catalyzed imine bond cleavage. The detailed characterization results of the BCPs are summarized in Table 5. The conventional BCP PS_28k_-*b*-PMMA_28k_, with a PS volume fraction (ƒ_St_) close to 50%, forms defect-free, fingerprint-like lamellar morphologies over an area of ≈ 0.95 µm^2^ on all four neutral mats (Mats 63–72). The resulting patterns exhibit low contrast after wet etching without UV exposure. In contrast, the acid-cleavable BCP system PS-N=CH-PMMA exhibits a gradual decrease in ƒ_St_ from 61.3% to 51.2% as the PMMA block length increases from 23k to 30k, accompanied by a morphological transition from coexisting cylindrical and lamellar phases to predominantly lamellar structures. Concurrently, the domain spacing (*L*_0_) increases from 36.1 nm to 40.2 nm, while the domain width of PMMA expands from 13.3 nm to 18.2 nm. These morphological evolutions demonstrate the tunability of microdomain geometry via PMMA block design, which is critical for optimizing etch contrast and pattern fidelity.

**Figure 4:**
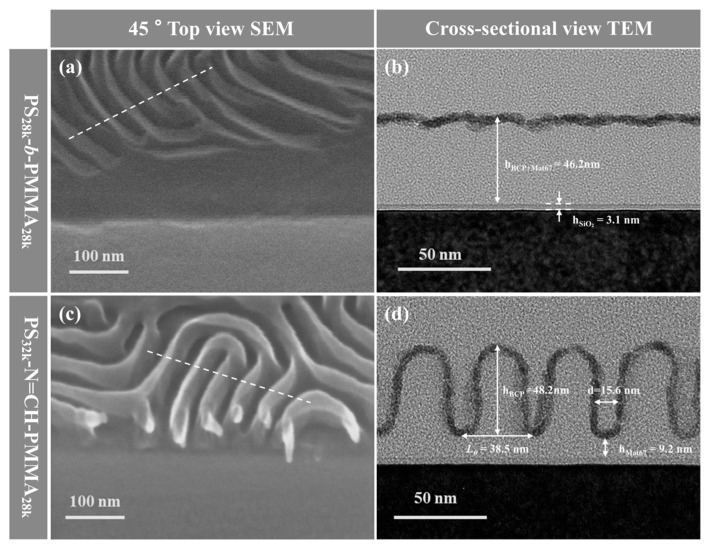
Top-view SEM images tilted by 45° and FIB-TEM cross-sectional images of lamella-forming BCP thin films, PS_28k_-*b*-PMMA_28k_ and PS_32k_-N=CH-PMMA_28k_, after thermal annealing on Mat67 and subsequent wet etching. (**a**,**b**) Top-view SEM (**a**) and cross-sectional TEM (**b**) images of PS_28k_-*b*-PMMA_28k_ thin film. (**c**,**d**) Top-view SEM (**c**) and cross-sectional TEM (**d**) images of PS_32k_-N=CH-PMMA_28k_ thin film. The dotted white lines in panels (**a**,**c**) indicate the positions where the cross-sectional TEM images in panels (**b**,**d**) were taken.

To assess the vertical etching behavior of lamella-forming BCPs under UV-free wet development, cross-sectional SEM and FIB-TEM analyses were conducted on PS_28k_-*b*-PMMA_28k_ and PS_32k_-N=CH-PMMA_28k_ films after thermal annealing and subsequent acetic acid treatment, as shown in Figure 4. To assess the vertical etching behavior of lamella-forming BCPs under UV-free wet development, we performed cross-sectional SEM and FIB-TEM analyses. The specimens were PS_28k_-*b*-PMMA_28k_ and PS_32k_-N=CH-PMMA_28k_ films subjected to thermal annealing followed by acetic acid treatment. Representative micrographs are presented in Figure 4. For the conventional PS_28k_-*b*-PMMA_28k_ system, the top-view SEM image (Figure 4a) reveals a blurred fingerprint-like morphology, and the cross-sectional TEM (Figure 4b) shows no clear domain contrast, indicating poor PMMA removal and the absence of vertical PS features. This result is consistent with the weak etching responsiveness observed in Figure 3b and highlights the limitation of covalently linked PS-*b*-PMMA in UV-free wet development. In contrast, the acid-cleavable PS_32k_-N=CH-PMMA_28k_ film exhibits a well-defined lamellar morphology after etching. As shown in Figure 4c,d, the PMMA domains were completely removed after wet etching, while the continuous PS phase remained intact, forming well-defined features with a thickness of approximately 48.2 nm. The interline spacing corresponding to the removed PMMA regions was measured to be 15.6 nm. The etch front reached the interface of the underlying neutral mat layer (h_Mat67_ ≈ 9.2 nm), further confirming the high selectivity of PMMA removal enabled by the acid-cleavable block junction. This enhanced etch profile is attributed to the cleavage of the imine (-N=CH-) linkage, which decouples the PS and PMMA blocks and enables efficient dissolution of the detached PMMA domains. Collectively, these results demonstrate that the cleavable junction enhances the chemical responsiveness of the PMMA block. They also show that it preserves the dimensional fidelity of the PS nanostructure under UV-free, mild etching conditions.

### 3.3. Graphoepitaxial DSA and Wet Etching Behavior of Lamella-Forming PS-N=CH-PMMA on Trench Templates

To investigate the self-assembly behavior of the synthesized lamella-forming PS-N=CH-PMMA BCPs on graphoepitaxial templates and the influence of block composition on line/pattern modulation, trench templates with well-defined structural parameters were fabricated using 193i lithography, following the procedure reported by Guillaume et al. [60]. The trench critical dimension (CD) was fixed at 55 nm, while the pitch varied from 160 to 240 nm. When the trench width closely matched the natural periodicity (*L*_0_) of the BCP, a single lamellar line was confined within each trench [61]. This two-line, one-space-per-trench configuration represents a key objective of this study, as it demonstrates precise graphoepitaxial guidance and provides a viable route toward sub-30 nm line spacing patterning with high fidelity. Maintaining constant lateral confinement while varying the trench pitch is a widely adopted strategy for regulating the fill depth of BCPs [62]. By adjusting the trench density on guiding templates, the fill depth can be effectively tailored to accommodate different PS mask thickness requirements in practical applications.

**Figure 5:**
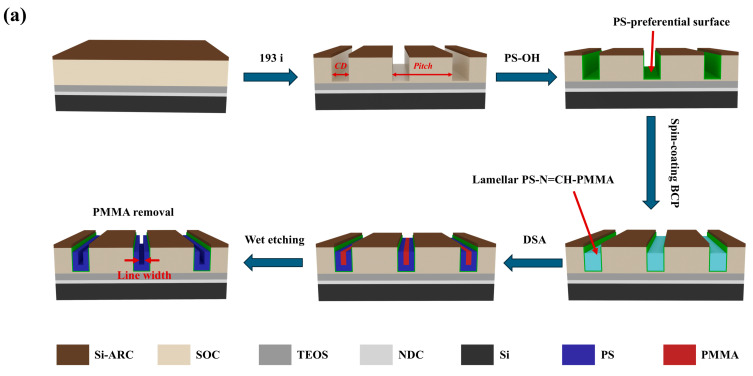

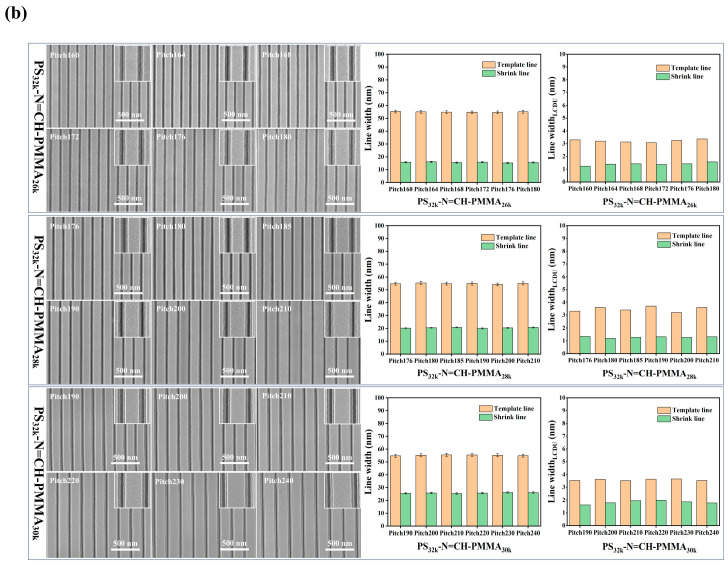
Morphological analysis of PS-N=CH-PMMA BCPs after graphoepitaxial DSA and wet etching. (**a**) Schematic illustration of the graphoepitaxial DSA process using lamella-forming PS-N=CH-PMMA BCPs in 193i-fabricated trench templates with a critical dimension (CD) of 55 nm. A PS-preferential brush was grafted to sidewalls, followed by spin coating, thermal annealing, and selective removal of PMMA domains to generate PS line patterns. (**b**) Top-view SEM images, including large-area overviews and locally magnified views (0.61 μm × 0.61 μm), along with corresponding bar plots showing average line widths and line width critical dimension uniformity (line width _LCDU_) for PS_32k_-N=CH-PMMA_26k_, PS_32k_-N=CH-PMMA_28k_, and PS_32k_-N=CH-PMMA_30k_ under varying pitch conditions (160–240 nm). Line width and line width _LCDU_ values were measured after wet etching (n = 20).

As shown in Figure 5a, graphoepitaxial DSA was conducted on the trench templates in which the trench sidewalls and bottom surfaces were grafted with PS-OH brushes to establish a PS-preferential wetting interface. Following surface modification, the BCP was spin-coated onto the trench template and subsequently subjected to thermal annealing to induce the formation of lamellar structures aligned parallel to the trench direction. Thereafter, wet etching with glacial acetic acid for 5 min was conducted to selectively remove the PMMA domains, resulting in well-defined PS line patterns. Notably, the interline space width was significantly reduced compared to the original trench critical dimension of 55 nm, demonstrating a line spacing shrinkage effect analogous to the hole shrink process observed in cylindrical confinement [63]. Figure 5b presents high-resolution SEM images of the DSA line/space patterns obtained after wet etching of three PS-N=CH-PMMA copolymers (PS_32k_-N=CH-PMMA_26k_, PS_32k_-N=CH-PMMA_28k_, and PS_32k_-N=CH-PMMA_30k_), which share a fixed PS block length (32k) and differ in PMMA block lengths (26k, 28k, and 30k). All three materials yielded well-aligned sub-30 nm line spacing across the entire pitch range, confirming the feasibility of this acid-cleavable lamellar system for nanoscale pattern miniaturization. The interline space width of the resulting shrink patterns increased monotonically with PMMA block length, ranging from approximately 15 nm for PS_32k_-N=CH-PMMA_26k_ to approximately 25 nm for PS_32k_-N=CH-PMMA_30k_. This trend is consistent with previous reports on conventional PS-*b*-PMMA systems, where longer PMMA chains promote lateral PMMA domain expansion during microphase separation, leading to wider interline space features [64]. Notably, all samples exhibited excellent dimensional control, with the line spacing local critical dimension uniformity (line width _LCDU_) values below 2.0 nm—substantially lower than that of the original trench CD (~3.2 nm). These results underscore the high pattern fidelity exhibited by the PS-N=CH-PMMA BCP series under graphoepitaxial confinement, achieved through direct wet etching.

**Figure 6:**
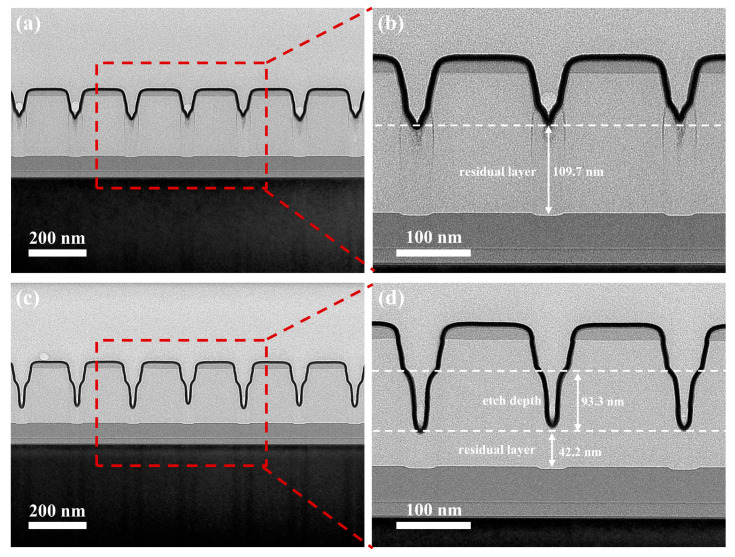
Cross-sectional TEM images of PS_28k_-*b*-PMMA_28k_ and PS_32k_-N=CH-PMMA_28k_ after graphoepitaxial DSA and subsequent wet etching. (**a**) Cross-sectional TEM images of PS_28k_-*b*-PMMA_28k_ assembled in trench templates (CD = 50 nm, pitch = 190 nm). (**b**) Magnified view of the region highlighted in (**a**). (**c**) Cross-sectional TEM images of PS_32k_-N=CH-PMMA_28k_ assembled in trench templates (CD = 55 nm, pitch = 190 nm). (**d**) Magnified view of the region highlighted in (**c**).

To further assess vertical wet etching responsiveness and structural fidelity, a comparative FIB-TEM analysis was performed between PS_32k_-N=CH-PMMA_28k_ and conventional PS_28k_-*b*-PMMA_28k_. Figure S9 shows top-view SEM images of PS_28k_-*b*-PMMA_28k_ after graphoepitaxial DSA on trench templates with a CD of 50 nm and trench pitches ranging from 190 nm to 240 nm, subjected to identical DSA processing conditions. Under these conditions, PS_28k_-*b*-PMMA_28k_ produced shallow etched features with substantial polymer residue remaining at the trench bottom (~109.7 nm) in the absence of UV pretreatment, indicating insufficient vertical separation between PMMA and PS blocks (Figure 6a,b). In contrast, PS_32k_-N=CH-PMMA_28k_ (Figure 6c,d) exhibited a greater etch depth (~93.3 nm) and significantly reduced residue (~42.2 nm), demonstrating enhanced wet etch selectivity. A tapered PS mask profile was observed near the trench bottom, which is likely attributable to two contributing factors: (1) the lack of interfacial neutrality, leading to preferential PS wetting and chain accumulation at the base; and (2) slight angular deviations in the trench sidewalls, which promote asymmetric chain packing and further intensify PS enrichment at the bottom [52]. Notably, after wet etching, PS-N=CH-PMMA-based self-assembled (SA) patterns exhibit higher PS structural stability compared to density-multiplied line/space patterns formed via graphoepitaxial DSA. In the case of PS-N=CH-PMMA thin films processed under self-assembly (SA) conditions (Figure 3b), the film thickness is relatively low (≤1.25 *L*_0_), and the resulting PS matrix exhibits a fingerprint-like morphology after PMMA removal, without evidence of capillary collapse. The thinner film thickness leads to lower structural height and diminished capillary torque, thereby improving the mechanical stability of the residual PS nanostructures during wet processing [65]. In contrast, the BCP film confined within the trench during graphoepitaxial DSA is substantially thicker (≥3 *L*_0_), which increases its susceptibility to capillary-induced bending stress during PMMA removal. When the trench critical dimension (CD) closely matches the natural periodicity (*L*_0_), a single PMMA domain is formed within each trench, and the adjacent PS lamellae are laterally anchored to the trench sidewalls. This geometric confinement provides mechanical stabilization, resulting in high-fidelity pattern retention after wet etching. Under density-multiplied conditions (e.g., 5× or 4× lines per trench), the PS lines are more closely spaced and less laterally supported (as shown in Figures S10 and S11) [66]. During PMMA removal, capillary forces acting between adjacent PS lines induce lateral attraction, frequently leading to pattern collapse and bridging [67]. This analysis shows that PS-N=CH-PMMA enables UV-free wet etching in both SA and DSA configurations. Importantly, the structural integrity of DSA-derived line/space patterns is strongly dependent on line spacing and trench confinement.

### 3.4. Ohta–Kawasaki Model Simulation of Trench Sidewall Angle Effects on the Graphoepitaxial DSA of Lamellar PS_32k_-N=CH-PMMA_28k_

**Figure 7:**
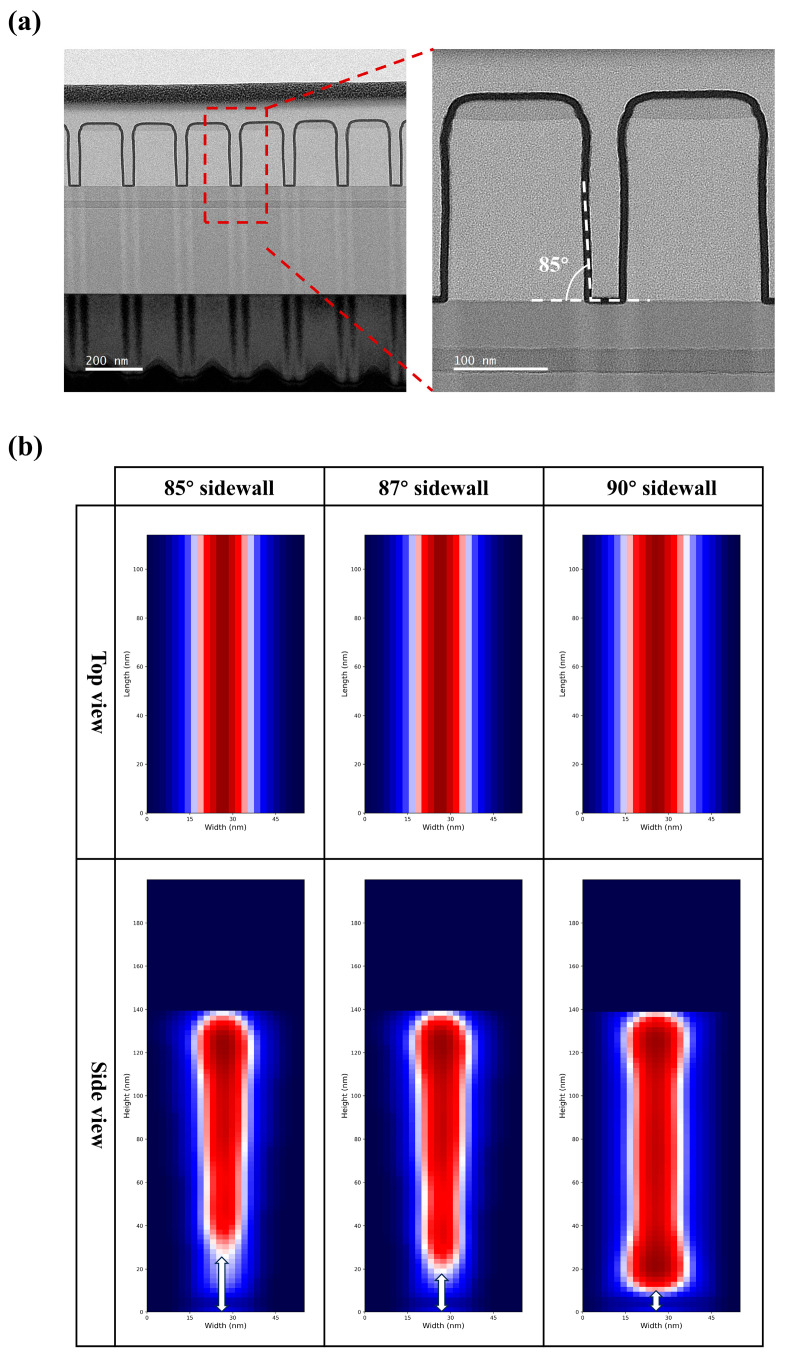
Effect of trench sidewall angle on the directed self-assembly of lamella-forming BCP PS_32k_-N=CH-PMMA_28k_ simulated by the Ohta–Kawasaki model. (**a**) Cross-sectional TEM images of trench array guiding templates with non-vertical sidewalls. The magnified view highlights a sidewall angle of 85°. (**b**) Ohta–Kawasaki simulations of BCP directed self-assembly within trench templates with sidewall angles of 85°, 87°, and 90°. Top-view (**top row**) and side-view (**bottom row**) profiles show the evolution of PS and PMMA domain morphology under varying confinement geometries. PS domains are shown in blue, and PMMA in red.

The trench sidewall angle of guiding templates is a critical yet often overlooked parameter that significantly influences the pattern morphology and fidelity of graphoepitaxial DSA. To elucidate its influence on the morphology of cleavable lamellar BCPs, we employed the OK model to simulate PS_32k_-N=CH-PMMA_28k_ behavior in trench templates with different trench sidewall angles (85°, 87°, and 90°). As shown in Figure 7a, cross-sectional TEM images reveal that the fabricated templates possess slightly tapered trench sidewalls (~85°), which motivated the modeling study. Figure 7b presents top- and side-view morphologies of PS (blue) and PMMA (red) domains under varying geometric confinements. All cases exhibited vertically aligned lamellae. As the trench sidewall angle decreased from 90° to 85°, the PS domains increasingly accumulated near the trench bottom, exhibiting pronounced tapering and distortion. This trend likely results from geometric frustration induced by narrowed trench bottoms, which locally constrain polymer chain extension and promote asymmetric wetting. These effects are particularly pronounced under high aspect ratio confinement, where vertical symmetry is essential for maintaining lamellar uniformity. Moreover, the simulated PS enrichment near the trench base provides a rational explanation for the tapered PS profile and the ~42.2 nm polymer residue observed in the cross-sectional FIB-TEM images after wet etching (Figure 6d). Therefore, achieving nearly vertical trench sidewall profiles is crucial for preserving DSA pattern integrity and minimizing etch-induced PS residues, especially in UV-free wet etching systems using cleavable PS-N=CH-PMMA BCPs.

## 4. Conclusions

In summary, we synthesized a series of acid-cleavable, lamella-forming PS-N=CH-PMMA BCPs featuring dynamic Schiff base linkages for sub-30 nm line spacing patterning. These materials preserve the thermal processability of conventional PS-*b*-PMMA systems, while enabling the formation of vertically aligned fingerprint-like patterns and facilitating graphoepitaxial directed self-assembly under standard annealing conditions. The interline space within a fixed one-space-per-trench configuration was tunable from 15 to 25 nm by adjusting the PMMA block length. A direct or UV-free wet etching strategy successfully removed the PMMA domains in a single step, yielding high-fidelity PS masks with significantly improved etch selectivity and reduced residue compared to PS-*b*-PMMA. Under graphoepitaxial confinement, trench sidewall tapering led to asymmetric PS accumulation near the trench bottom, which remained resistant to complete removal and limited the vertical etch depth to ~93.3 nm. This morphology was corroborated by Ohta–Kawasaki simulations, confirming the geometric origin of residual features. Overall, this study confirms the feasibility of leveraging dynamic covalent chemistry to improve the wet etching capability of BCP lithography and presents a thermally compatible, UV-free wet development strategy for high-resolution nanopatterning in semiconductor fabrication.

## Figures and Tables

**Table 1:** GPC characterization results of PMMA-OH samples with different molecular weights.

Samples	M_p_ (kg/mol)	M_n_ (kg/mol)	M_w_ (kg/mol)	PDI	DP_n_
PMMA_23k_-OH	25.3	23.0	25.1	1.09	230
PMMA_26k_-OH	27.2	25.9	27.5	1.09	259
PMMA_28k_-OH	31.3	28.2	30.7	1.09	282
PMMA_30k_-OH	32.6	30.1	32.8	1.09	301

**Table 2:** GPC results of PMMA-CHO samples with different molecular weights.

Samples	M_p_ (kg/mol)	M_n_ (kg/mol)	M_w_ (kg/mol)	PDI	GE (%)
PMMA_23k_-CHO	25.5	23.1	25.2	1.09	87.3
PMMA_26k_-CHO	27.7	26.1	28.4	1.09	86.1
PMMA_28k_-CHO	31.9	28.3	31.2	1.09	85.9
PMMA_30k_-CHO	33.6	30.3	33.1	1.09	84.2

**Table 3:** GPC results and block compositions of PS-b-PMMA and PS-N=CH-PMMA copolymers with varying PMMA block lengths.

Samples	M_p_ (kg/mol)	M_n_ (kg/mol)	M_w_ (kg/mol)	PDI
PS_28k_-*b*-PMMA_28k_	64.8	56.3	61.9	1.10
PS_32k_-N=CH-PMMA_23k_	65.2	55.3	62.5	1.13
PS_32k_-N=CH-PMMA_26k_	68.0	58.0	65.6	1.13
PS_32k_-N=CH-PMMA_28k_	70.2	60.1	68.0	1.13
PS_32k_-N=CH-PMMA_30k_	71.9	61.7	69.7	1.13

**Table 4:** GPC results and styrene molar fractions of random copolymer poly (St-co-MMA-co-GMA).

Samples	M_p_ (kg/mol)	M_n_ (kg/mol)	M_w_ (kg/mol)	PDI	F_St_ (%)	Film Thickness (nm)
Mat63	39.2	24.1	37.4	1.55	63.2	7.8
Mat65	41.3	25.1	39.4	1.57	65.4	8.5
Mat67	44.1	28.1	43.8	1.56	67.1	9.7
Mat72	45.6	28.5	44.2	1.55	72.3	10.2

**Table 5:** Characterization of PS-*b*-PMMA and PS-N=CH-PMMA BCPs.

Samples	M_n_ of PMMA ^a^ (kg/mol)	ƒ_St_^b^ (%)	Domain Width of PMMA ^c^ (nm)	*L*_0_ ^d^ (nm)	Film Thickness ^e^ (nm)
PS_28k_-*b*-PMMA_28k_	28.1	51.2	12.9	29.8	38.3
PS_32k_-N=CH-PMMA_23k_	23.1	61.3	-	-	-
PS_32k_-N=CH-PMMA_26k_	26.1	57.2	13.3	36.1	45.1
PS_32k_-N=CH-PMMA_28k_	28.3	54.8	15.4	38.3	47.9
PS_32k_-N=CH-PMMA_30k_	30.3	52.4	18.2	40.2	50.2

^a^ M_n_ of the PMMA was determined by GPC. ^b^ ƒ_St_ was calculated by ^1^H NMR. ^c^ PMMA domain width was measured from SEM analysis of BCP thin films self-assembled on the neutral substrate Mat67. ^d^ Domain spacing (*L*_0_) was obtained by SEM analysis of BCP thin films self-assembled on the neutral substrate Mat67. ^e^ Film thickness was measured using a Filmetrics F20-UV spectroscopic reflectometer.

## Data Availability

The data that support the findings of this study are available on request from the corresponding author.

## Supplementary Materials

The following supporting information can be downloaded at: https://www.mdpi.com/article/10.3390/polym17182435/s1, Figure S1: ^1^H NMR spectra of PS-N=CH-PMMA BCPs with different PMMA block lengths and conventional PS-*b*-PMMA. Figures S2 and S3: GPC traces of hydroxyl- and aldehyde-terminated PMMA precursors with molecular weights of 23k, 26k, 28k, and 30k. Figure S4: GPC traces of PS-N=CH-PMMA BCPs. Figure S5: GPC traces of conventional PS-*b*-PMMA and the macroinitiator PMMA_28k_-Br. Figure S6: Synthetic scheme of epoxy-functional random copolymer Mats. Figures S7 and S8: ^1^H NMR spectra and GPC traces of Mats with different styrene molar fractions (F_St_). Figure S9: Top-view SEM images of PS-*b*-PMMA after graphoepitaxial DSA and wet etching in trench templates (CD = 50 nm; pitch = 190–240 nm). Figures S10 and S11: Top-view SEM images of PS-*b*-PMMA and PS-N=CH-PMMA assembled in trench templates (width = 135–150 nm; pitch = 300 nm), exhibiting fourfold and threefold density multiplication, respectively.
