# Supplementary material for: An Acid-Cleavable Lamellar Block Copolymer for Sub-30-nm Line Spacing Patterning via Graphoepitaxial Directed Self-Assembly and Direct Wet Etching
Source: Polymers (Basel). 2025 Sep 9;17(18):2435. doi: 10.3390/polym17182435 (PMC12473877; doi:10.3390/polym17182435)
Supplement: Supplementary file 1 [file polymers-17-02435-s001.zip › polymers-3825651-supplementary.pdf]

Supplementary Information

An Acid-Cleavable Lamellar Block Copolymer for Sub-30 nm Line Spacing Patterning via Graphoepitaxial Directed Self-Assembly and Direct Wet Etching

*Jianghao Zhan [1], Caiwei Shang [1], Muqiao Niu [1], Jiacheng Luo [1], Shengguang Gao [2], Zhiyong Wu [2], Shengru Niu [1], Yiming Xu [1], Xingmiao Zhang [2], Zili Li [1], and Shisheng Xiong [1,2,]**

1. Center of Micro-Nano System, School of Information Science and Technology, Fudan University, Shanghai, 200438, China

2. Zhangjiang Laboratory, 100 Haike Road, Shanghai, 201204, China

Corresponding Author

* E-mail address: sxiong@fudan.edu.cn.

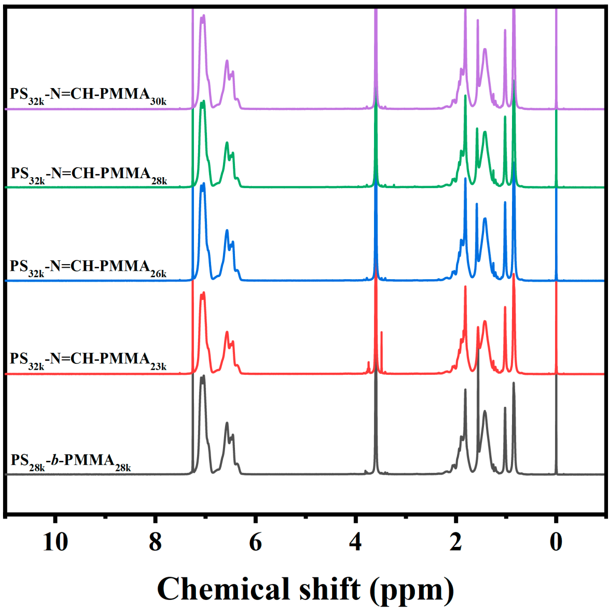

Figure S1. ^{1}H NMR of acid-cleavable block copolymers PS_{32k}-N=CH-PMMA with varying PMMA block molecular weights (23k, 26k, 28k, and 30k), along with a conventional block copolymer PS_{28k}-*b*-$PMMA_{28k}$.

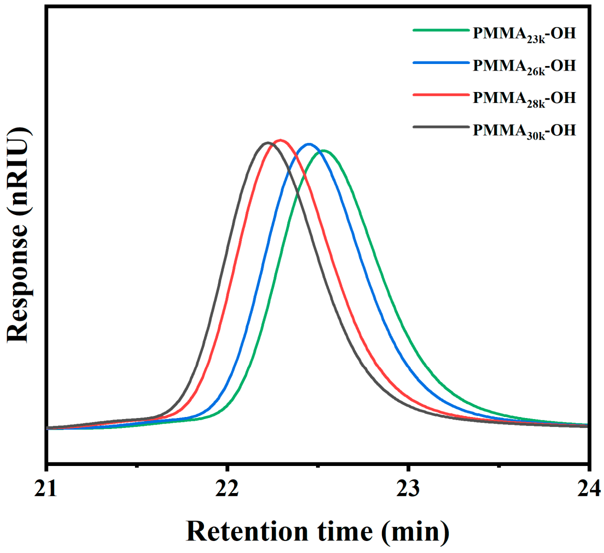

Figure S2. GPC traces of hydroxyl-terminated poly (methyl methacrylate) (PMMA-OH) with molecular weights of 23k, 26k, 28k, and 30k.

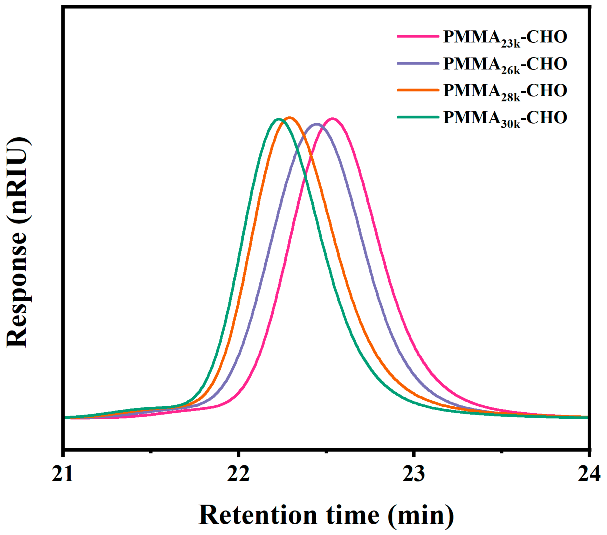

Figure S3. GPC traces of aldehyde-terminated poly (methyl methacrylate) (PMMA-CHO) with molecular weights of 23k, 26k, 28k, and 30k.

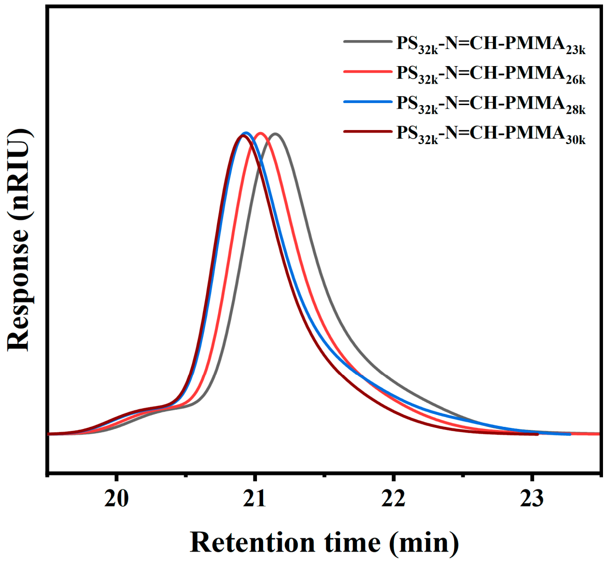

FigureS4. GPC traces of acid-cleavable block copolymers PS_{32k}-N=CH-$PMMA_x$ (x= 23k, 26k, 28k, and 30k).

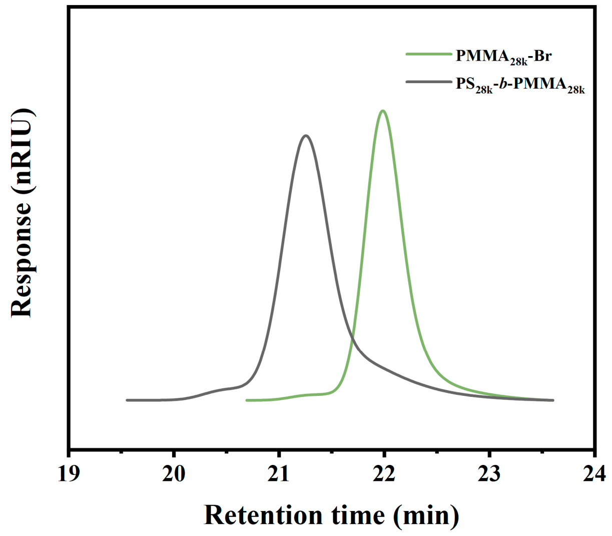

Figure S5. GPC traces of conventional block copolymer PS_{28k}-*b*-$PMMA_{28k}$ and the PMMA macroinitiator $PMMA_{28k}$-Br.

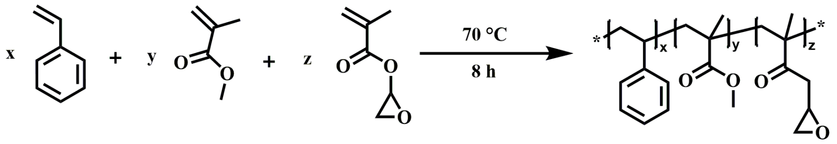

FigureS6. Synthesis scheme of random copolymers Poly(St-r-MMA-r-GMA) (Mats) with pendant epoxy groups.

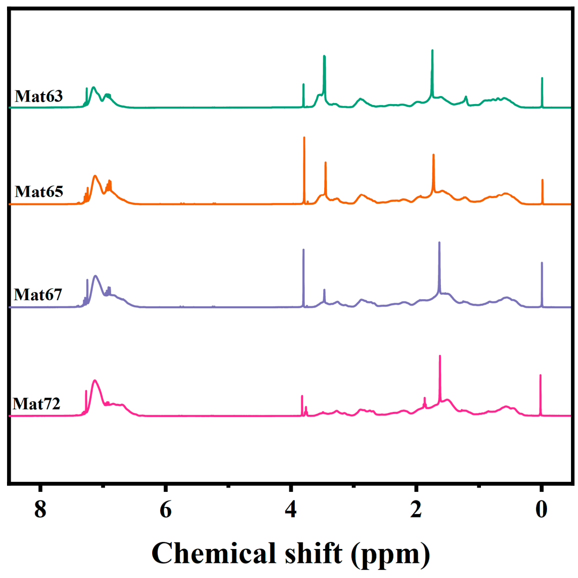

Figure S7. ^{1}H NMR spectra of random copolymers poly (St-r-MMA-r-GMA) with different styrene feed molar fractions (F_{St}), where Mat63, Mat65, Mat67, and Mat72 correspond to $F_{St} = 0.63$, 0.65, 0.67, and 0.72, respectively.

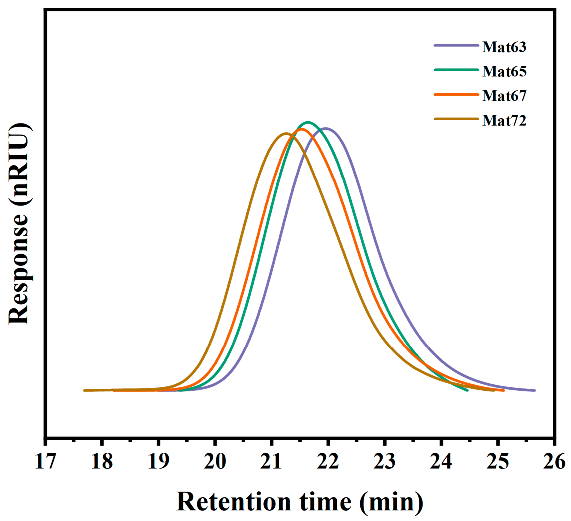

Figure S8. GPC traces of random copolymers poly (St-r-MMA-r-GMA) with different styrene feed molar fractions (F_{St}).

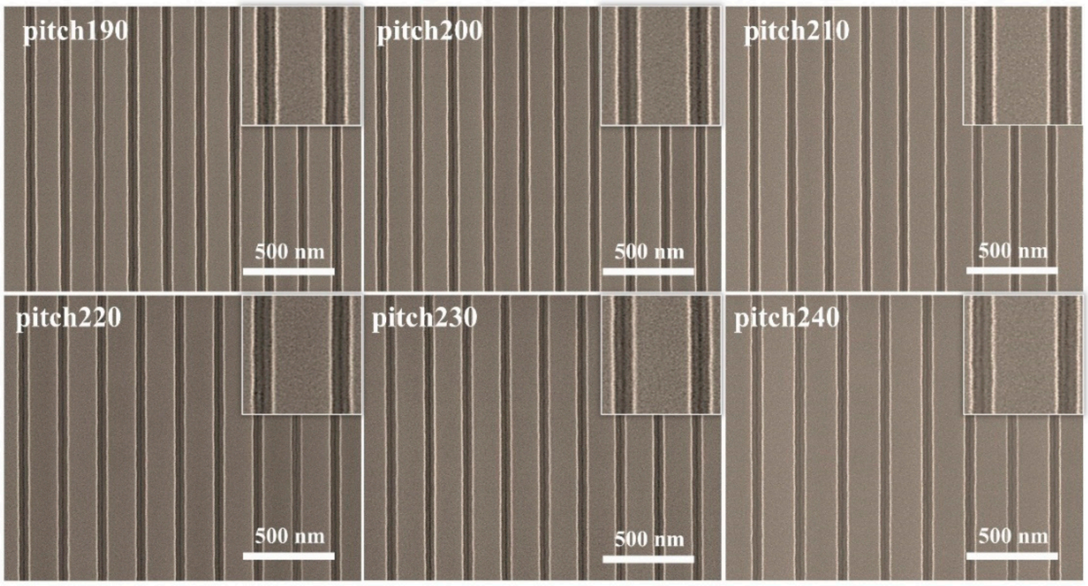

Figure S9. Top-view SEM images of block copolymer PS_{28k}-*b*-$PMMA_{28k}$ after graphoepitaxial DSA on guiding templates with CD50nm and trench pitches ranging from 190 nm to 240 nm and subsequent wet etching.

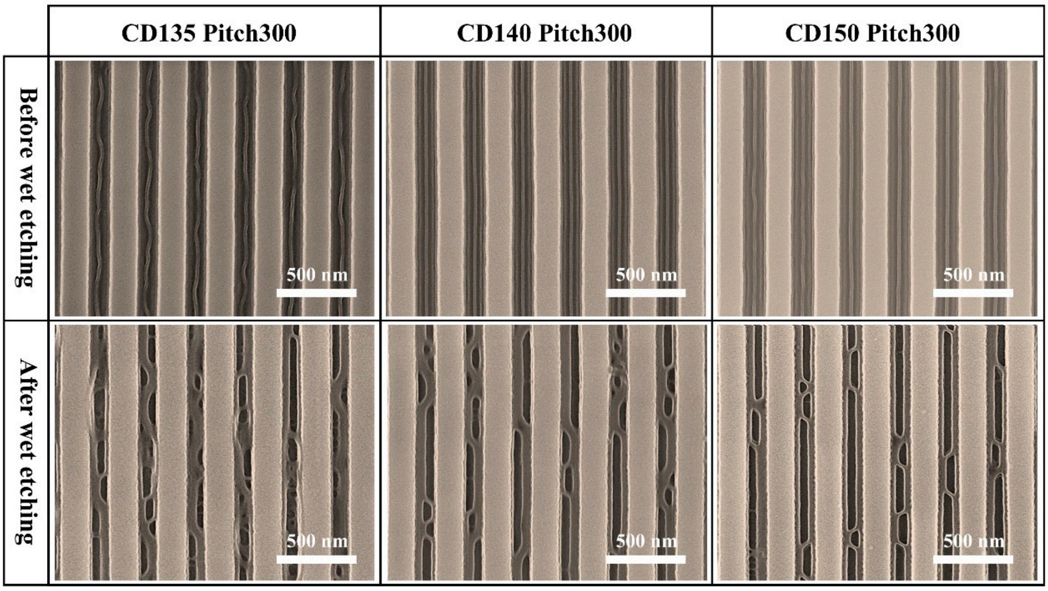

Figure S10. Top-view SEM images of PS_{28k}-*b*-$PMMA_{28k}$ directed self-assembled in trench templates (widths: 135–150 nm; pitch: 300 nm), exhibiting fourfold density multiplication before and after wet etching.

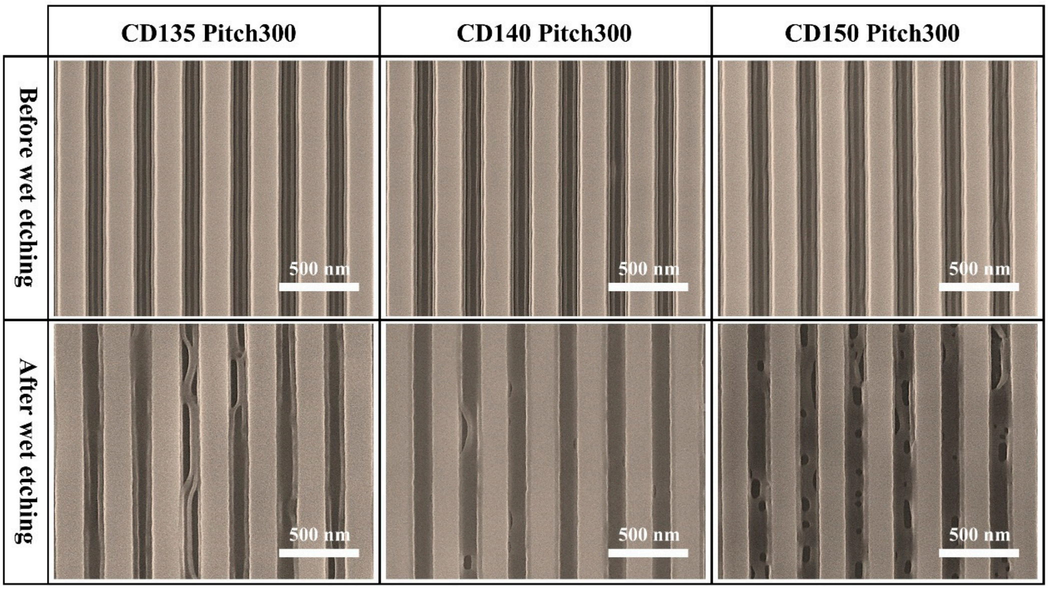

Figure S11. Top-view SEM images of PS_{32k}-N=CH-$PMMA_{28k}$ directed self-assembled in trench templates (widths: 135–150 nm; pitch: 300 nm), exhibiting threefold density multiplication before and after wet etching.
